# Effects of Transpulmonary Administration of Caffeine on Brain Activity in Healthy Men

**DOI:** 10.3390/brainsci9090222

**Published:** 2019-09-03

**Authors:** Kazutaka Ueda, Masayuki Nakao

**Affiliations:** Department of Mechanical Engineering, Graduate School of Engineering, The University of Tokyo, 7-3-1 Hongo, Bunkyo-ku, Tokyo 113-8656, Japan

**Keywords:** caffeine, transpulmonary administration, working memory, electroencephalography, prefrontal cortex

## Abstract

The present study aimed to examine the effect of transpulmonary administration of caffeine on working memory and related brain functions by electroencephalography measurement. The participants performed working memory tasks before and after vaporizer-assisted aspiration with inhalation of caffeinated- and non-caffeinated liquids in the caffeine and sham conditions, respectively. Transpulmonary administration of caffeine tended to increase the rate of correct answers. Moreover, our findings suggest that transpulmonary administration of caffeine increases the theta-band activity in the right prefrontal, central, and temporal areas during the task assigned post-aspiration. Our results may indicate an efficient and fast means of eliciting the stimulatory effects of transpulmonary administration of caffeine.

## 1. Introduction

In recent years, vaporizers have been widely used to gain exhilaration and improve cognitive function. The vaporizer is a device that atomizes liquid by the heat generated from the heating element and performs transpulmonary aspiration of caffeine and herbs, besides nicotine. Oral consumption of caffeine has been reported to improve vigilance [1,2], attention [3,4], memory function [5], and mood [6]. On the contrary, little is known about the effects of transpulmonary administration of caffeine on cognitive function. When administered orally, the peak blood level of caffeine is achieved in 30 to 120 min [7], whereas transpulmonary administration achieves the same in a few seconds [8]. Moreover, caffeine is known to pass through the brain-blood barrier [9]. Based on these facts, transpulmonary administration of caffeine can be expected to have an immediate effect in improving cognitive and related brain functions.

The purpose of this study was to investigate the effects of transpulmonary administration of caffeine on cognitive and related brain functions. We performed electroencephalography (EEG) measurements of participants performing a working memory task before and after the transpulmonary administration of caffeine. We analyzed brain activity in the theta band [10], which is suggested to be related to working memory functions.

## 2. Materials and Methods

### 2.1. Participants

Nine healthy male participants (mean age ± standard deviation: 22.8 ± 1.4 years) with normal or corrected-to-normal vision participated in the experiments. None of them had a history of neurological or psychiatric illness. All participants reported being low caffeine consumers (mean consumption = 75 mg/day), and non-smokers. All participants were right-handed, as determined by the Flinders handedness survey (FLANDERS) [11]. This study used a within-subject design to reduce error variance in the physiological measures and has sufficient statistical power to answer the research questions. The study protocol was approved by the Ethics Committee of the Graduate School of Engineering, the University of Tokyo. All participants provided written informed consent prior to their participation in this study.

### 2.2. Stimuli

We used a commercially available vaporizer, caffeinated liquid (caffeine 1%) for test, and non-caffeinated liquid for the sham condition. Both liquids were transparent and indistinguishable by appearance. Moreover, none of the liquids contained nicotine.

### 2.3. Experimental Task

**Figure 1:**
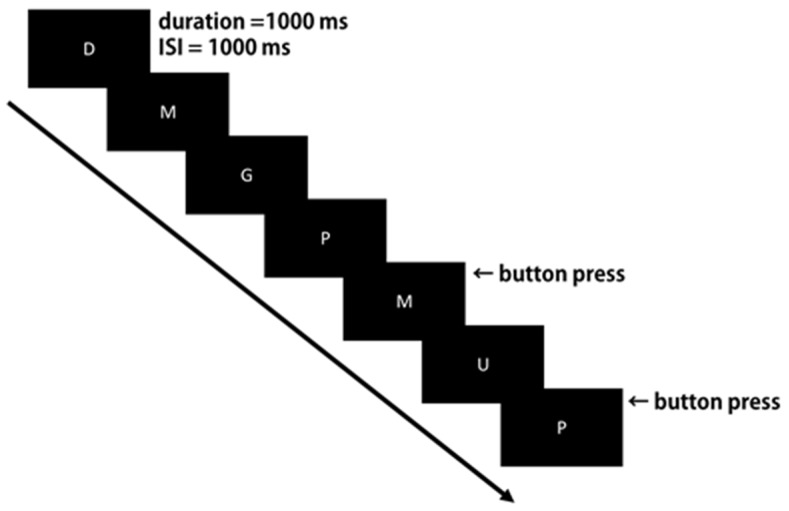
Experimental design of the letter 3-back working memory task.

The letter 3-back working memory tasks [12] were administered as neurobehavioral probes during EEG measurement. The sequences of the uppercase letters were centrally presented with a stimulus duration of 1000 ms and an interstimulus interval of 1000 ms against a black background using Presentation (Neurobehavioral Systems, Inc., Berkeley, CA, USA). Participants were required to press a button with their right finger immediately if the letter currently presented were the same as the previous three times (Figure 1).

### 2.4. Procedures

All participants underwent both caffeine and sham conditions at the same time on two separate days, with an interval of one or more days between the experimental days. The condition order was counterbalanced across participants.

The experiment took place in a shielded room with the participants seated in a comfortable chair, about 90 centimeters from a 29.8” type display MultiSync LCD-PA302W (NEC Corp, Tokyo, Japan; effective display area of 641 × 401 mm^2^). The participants were instructed to relax, prevent excessive body or head movements, and to fix their gaze on the middle of the monitor. After explaining the experiment, it was conducted in the following order while with the participants seated on a chair:

(1) Pre-aspiration task: The participants performed the letter 3-back working memory task for 4 min (120 trials).

(2) The participant performed vaporizer aspiration for 2 min.

(3) Post-aspiration task: The participant performed the letter 3-back working memory task for 4 min (120 trials).

The vaporizer aspiration was performed in eight sets with the following steps constituting a set:

(1) Suck steam for 2 s.

(2) Inhale deeply steam for 3 s.

(3) Exhale from his mouth for 6 s.

(4) Rest for 6 s.

The timings of these steps were indicated on the display. The caffeine content in the aspirated vapor in the caffeine condition was about 0.15 mg. Under either of the conditions (caffeine and sham), the participants were required to rate their subjective evaluation concerning the degree of preference and intensity of aroma on a 4-point scale immediately after vaporizer aspiration.

### 2.5. EEG Recording and Analysis

**Figure 2:**
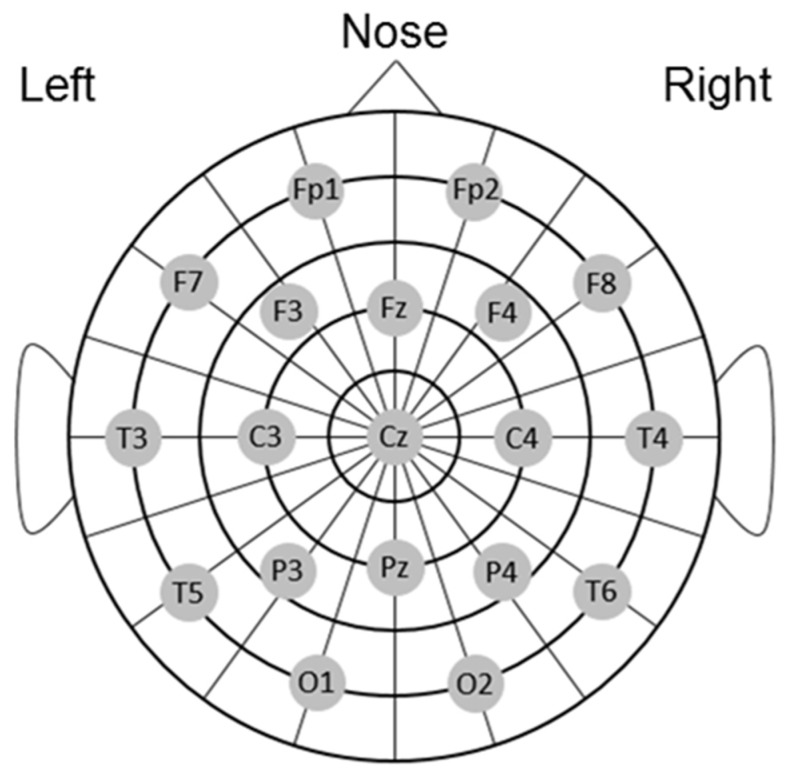
Electroencephalograph electrode positions (Electrode sites of the 10–20 system).

EEG signals were continuously recorded using the EEG-1200 (Nihon Kohden Corp., Tokyo, Japan) at a sampling rate of 1000 Hz. Nineteen electrodes were positioned according to the international 10−20 system for electrode placement (at the Fp1, Fp2, Fz, F3, F4, F7, F8, Cz, C3, C4, T3, T4, Pz, P3, P4, T5, T6, O1, and O2 sites; Figure 2) [13], using the average of both earlobes as reference, with a time constant of 10 s.

The continuous EEG data were segmented into 4-minute epochs, separately for the pre- and post-aspiration letter 3-back working memory task. The EEG data were exported to EEGLAB14.2b (MATLAB toolbox) [14] for spectral analysis, and were high-pass filtered at 1 Hz using a finite impulse response filter. Electrooculographic artifacts due to blinks or eye movements and electromyographic artifacts were removed using the Automatic Subspace Reconstruction method implemented in the ‘clean_rawdata’ plugin of EEGLAB [15]. To estimate the average power of the theta band (5–7 Hz), data were processed using the time-frequency algorithm in EEGLAB.

## 3. Results and Discussion

**Figure 3:**
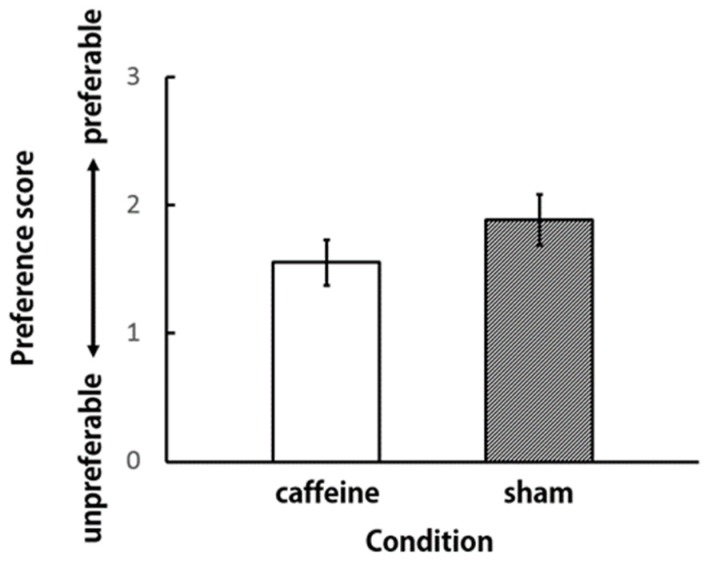
Preference score for the aroma of vapors (*N* = 9).

**Figure 4:**
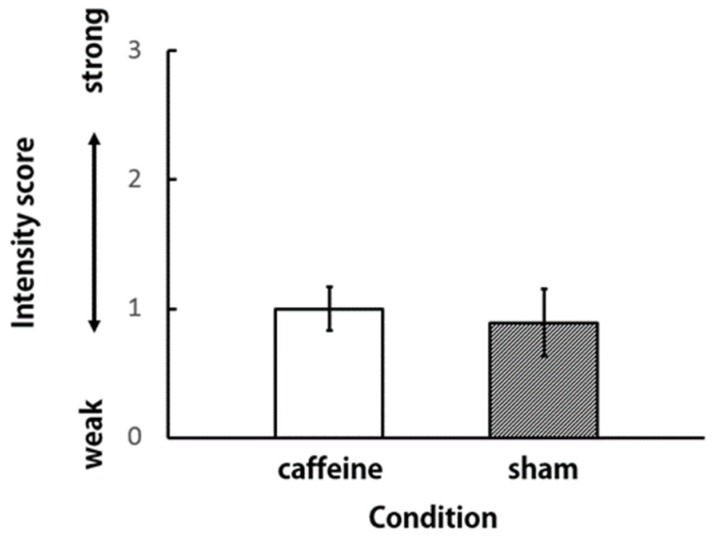
Intensity score for the aroma of vapors (*N* = 9).

In order to compare the participants’ impressions of the aroma of the vapors in the caffeine and sham conditions, the subjective preference and intensity for aroma were scored (Figure 3 and Figure 4). The paired t-test was performed with the score as the independent variable. There were no significant differences between scores in either of the conditions. We observed that the participants did not feel any difference in the aroma of the vapors under caffeine and sham conditions.

**Figure 5:**
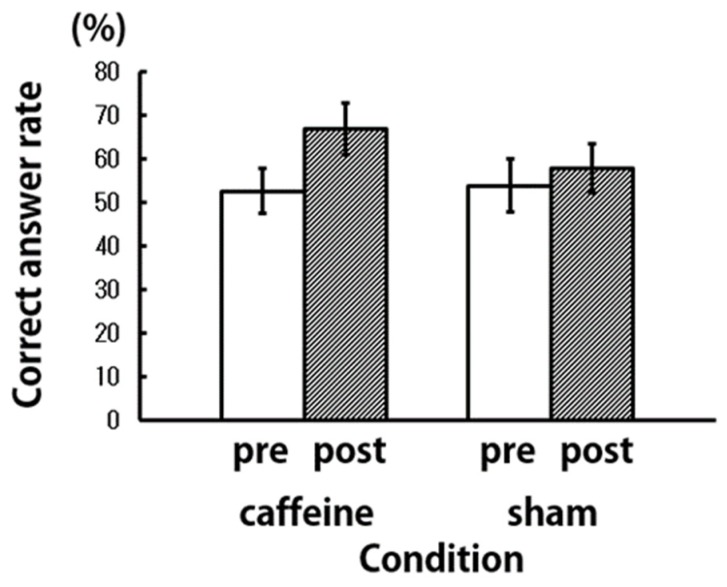
Percentage of correct answer in the letter 3-back working memory task (*N* = 9).

**Figure 6:**
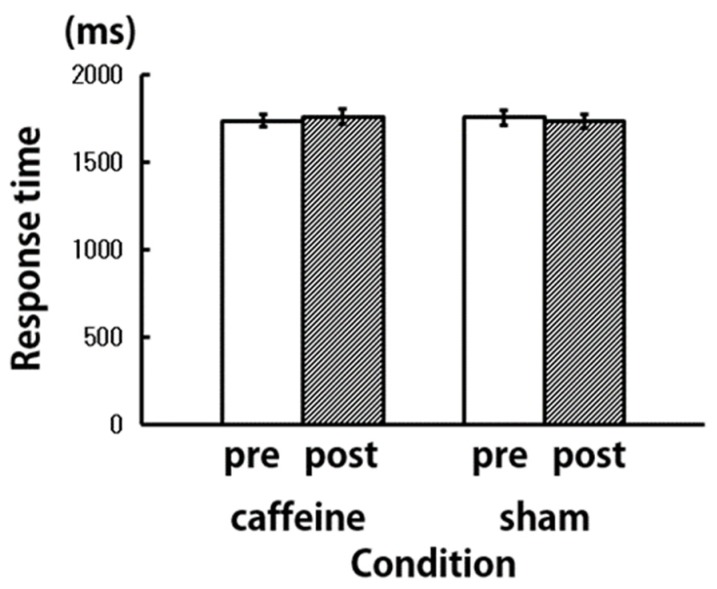
Response time of the letter 3-back working memory task (*N* = 9).

In order to compare the behavioral indices in the caffeine and sham conditions, the correct answer rate and the response time in the letter 3-back working memory task were calculated (Figure 5 and Figure 6). In the caffeine condition, the correct answer rate increased post-aspiration, compared to pre-aspiration. Even under the sham condition, the correct answer rate increased post-aspiration; however, this increase was lesser than that under the caffeine condition. There was no difference in the reaction time pre- and post-aspiration under both conditions. In a two-way repeated measures analysis of variance (ANOVA) using treatment (caffeine and sham) and time (pre- and post-aspiration) as the dependent variables, no significant differences were found in the correct answer rate and reaction time.

**Figure 7:**
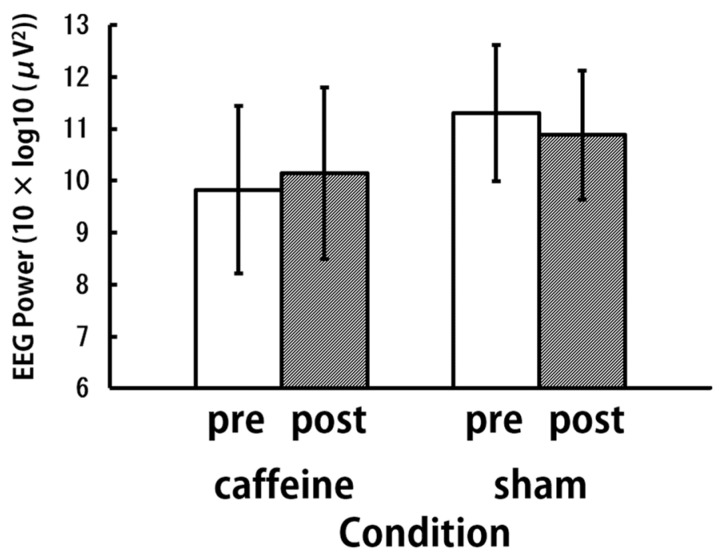
Theta activation at F8 during the letter 3-back working memory task (*N* = 9).

The brain activity in the theta band was calculated for a 4-minute epoch, separately for the pre- and post-aspiration letter 3-back working memory task. A two-way repeated measures ANOVA was performed with treatment (caffeine and sham) and time (pre- and post-aspiration) as the dependent variables, and the log-transformed (10 × log10 (μV^2^)) theta band power (5−7 Hz) as the independent variable. We observed significant interactions for F8, F4, C4, and T4 (*p* < 0.05) (Table 1, Figure 7). Theta-band power differences between post- and pre-aspiration during the letter 3-back working memory task in the caffeine and sham conditions were calculated. The averages of all nine participants in the experiment are presented in Table 1.

Activity of the right prefrontal cortex (F8 and F4), right central region (C4), and the right temporal region (T4) were enhanced after the aspiration of vapors in the caffeine condition as compared with the sham condition. These results are consistent with previously reported findings, which suggested that the activation of the right frontal area increased during the working memory task after oral administration of caffeine [16,17]. The neuroexcitatory action of caffeine, a non-selective adenosine A1 and A2 receptor antagonist, modulates the activity of the dopamine-rich brain regions of the right hemisphere that are involved in executive and attentional functions required for working memory function [16]. In the previous study, the participants performed the working memory task 20−30 min after oral administration of caffeine, whereas, in this study, the participants performed the task immediately after transpulmonary aspiration of caffeinated vapors. Our findings indicate that transpulmonary administration of caffeine has an immediate effect on the right prefrontal, central, and temporal areas associated with working memory.

With regard to the sham condition, the theta-band activities demonstrated greater deactivation in the post-aspiration task. Previous research has shown the relationship between the sustained effort to focus attention and theta-band activity under working memory load [18]. In the sham condition, it is possible that sustained effort decreased and the theta-band activity decreased with time. On the contrary, the increase in the brain activity for the theta band in the caffeine condition may indicate that caffeine contributes to sustain execution and attention.

In the caffeine condition, the post-aspiration correct answer rate increased more than that of the pre-aspiration. This increase was greater than that observed in the sham condition. However no significant differences were found in the behavioral response. This behavioral effect could be due to the low content of caffeine used in this study rather than the previous study [1,2,3,4,5,6]. However, as in this study, previous studies of brain function show significant changes in brain activity even without corresponding changes in overt behavior [16,19,20]. The effects of transpulmonary aspiration of caffeine on other brain activity related to resting state, vigilance, attention, and mood are not still clear and need further research.

## 4. Conclusions

The objective of this study was to investigate the effect of the transpulmonary administration of caffeine on working memory and related brain functions by EEG measurement. The participants performed the letter 3-back working memory tasks before and after vaporizer-assisted aspiration of caffeinated or sham liquid. The transpulmonary administration of caffeine tended to increase the rate of correct answers. Moreover, transpulmonary administration of caffeine was observed to immediately increase the theta-band activity in the right prefrontal, central, and temporal areas during task performance. These results may indicate an efficient and fast means of eliciting the stimulatory effects of transpulmonary administration of caffeine.

## Figures and Tables

**Table 1:** Theta-band power differences between post- and pre-aspiration during the letter 3-back working memory task in the caffeine and sham conditions (*N* = 9).

	Treatment Condition			
	Caffeine		Sham	
	ΔPower (Post–Pre)		ΔPower (Post–Pre)	
EEG Location	Mean	SE	Mean	SE
F8	0.314	0.225	−0.420	0.231
F4	0.164	0.168	−0.416	0.248
C4	0.173	0.171	−0.335	0.189
T4	0.290	0.199	−0.318	0.239
